# Strange, If True

**Published:** 1875-02

**Authors:** 


STRANGE, IF TRUE.—A remarkable story comes to us of the employment of a secret method of relieving sprained limbs by a family of French persons name Sequence, now living at Cohoes, New York. It is said that twelve years ago, the mother of Mrs. Sequence, who was then in France, imparted to her the secret of curing sprained limbs, that being all she professed to be able to do. It was a family secret, and had been handed down from generation to generation, and had never been known to fail. By means of it a complete cure can be effected, even in serious cases, although an essential point is that the attempt must be made soon after the injury is received. Mrs. Sequence could only cure men and boys, and in order to benefit both sexes the secret was imparted to her husband, who, strange to state, can only cure women and girls. They never take any pay for their services, believing that if they received fees the power of curing would be taken from them. They also believe that if the husband attempted to cure a man he would lose the power, and *vice versa* with the wife.

When asked to tell the *modus operandi*, the woman replied that that was her secret. No kind of liniment is employed, but she heals by laying her hands on the injured part, the patient being compelled to turn his or her head away during the performance of the operation. The cure is sometimes instantaneous, and sometimes gradual, but only one application is needed. People were incredulous, Mrs. S. said, but all who had ever been to either herself or husband had been convinced that they had the power. Kyran Egan, foreman of the card-room in the Ogden Mills at Cohoes, is reported to have said that some time ago he sprained his ankle so badly that he was compelled to remain in the house for more than a week. He was told by a daughter of Mrs. Sequence that her mother could cure him ; and although he had no faith in the statement, he visited her, being compelled to walk to the house with a cane. Upon arrival he was instructed to bare his foot and ankle. He did so, and Mrs. Sequence felt of it a few minutes, gently rubbing it with her hands, and then pronounced him cured. He felt no pain, stood upon the limb, found it all right, threw away his crutch, and has suffered no trouble from the sprain since. Kyran says he does not know how she did it, but she did it. Mr. Rivers, a brother of Officer Rivers, of Troy, had a sprained arm cured in a similar manner recently, and other cures are reported. These statements are singular, to say the least ; but the neighbors do not seem to doubt the credibility of these persons, and that the husband, wife, and son possess some peculiar powers is generally believed.

---

ENGLISH MARRIAGES.—In England and Wales, between 1861 and 1871, the number of males married at the age of 15, was, according to the offical statement, 35,206 ; of females, 104,998. There were more marriages at 20 than any other age ; the number of males at that age being 554,124, and of females, 569,317—to these being added 3,578 men and 5,136 women married a second time at that age. There were 77 cases in which the woman was 40 years older than her husband, and 38 cases in which the difference reached 60 years. The cases in which women have married men very much older than themselves were more numerous than the converse. To every 100 English married women between 15 and 55 there are born annually 22 children. In 1871, the number of marriages contracted was 190,112; ten years before, the number was 163,706. The average duration of married life is stated as 25 years.

